# 

**Published:** 1908-06-06

**Authors:** 


Telegraphic Address: Ospedale, London.

THE MODERN MEDICAL JOURNAL.

Telephone Number: 2734 Gerrard.

NEW SERIES. No. 64, Vol. III. No. 1136. Vol. XLIV.

SATURDAY, JUNE 6TH, 1908.

PRICE THREEPENCE.

